# Supplementary material for: Engineering cm-scale true push-pull electro-optic modulators in a suspended GaAs photonic integrated circuit platform by exploiting the orientation induced asymmetry of the Pockels r 41 coefficient
Source: Nanophotonics. 2025 Aug 28;14(18):3033–42. doi: 10.1515/nanoph-2025-0212 (PMC12442365; doi:10.1515/nanoph-2025-0212)
Supplement: Supplementary file 1 — Supplementary Material Details [file j_nanoph-2025-0212_suppl_001.pdf]

Research Article

Haoyang Li, Robert Thomas, Pisu Jiang, and Krishna C. Balram*

Engineering cm-scale true push-pull electro-optic modulators in a suspended GaAs photonic integrated circuit platform by exploiting the orientation induced asymmetry of the Pockels r_{41} coefficient

Abstract: Electro-optic modulators (EOMs) underpin a wide range of critical applications in both classical and quantum information processing. ~~While traditionally the focus has been on building these devices in materials with large Pockels coefficient (mainly ferroelectric insulators like lithium niobate), there is a need to engineer EOMs in a semiconductor platform with a view towards device stability (in radiation-hard environments), manufacturability (wafer size and foundry compatibility) and integration (with active electronics and quantum confined structures)~~. While these devices have been extensively optimized in a wide range of materials from ferroelectric insulators like lithium niobate to semiconductors like gallium arsenide and indium phosphide, there is a need to explore new design and manufacturing methods with a view towards improving device performance. Here, we demonstrate true push-pull EOMs in a suspended GaAs photonic integrated circuit (PIC) platform by exploiting the orientation induced asymmetry of the Pockels r_{41} coefficient, and folding the two arms of a cm-scale Mach-Zehnder interferometer (MZI) modulator along two orthogonal crystal axes. Our work also shows the potential of incorporating ideas from micro-electro-mechanical systems (MEMS) in integrated photonics by demonstrating high-performance active devices built around cm-scale suspended waveguides with sub-µm optical mode confinement.

Keywords: Electro-optic modulators, photonic integrated circuits, gallium arsenide, Pockels coefficient

Haoyang Li, Robert Thomas, Pisu Jiang, Krishna C. Balram, Quantum Engineering Technology Labs and School of Electrical, Electronic and Mechanical Engineering, University of Bristol, Woodland Road, Bristol BS8 1UB, United Kingdom,
***Corresponding author: Krishna C. Balram,** krishna.coimbatorebalram@bristol.ac.uk

1 Introduction

Electro-optic modulators (EOMs) are critical for mapping analog and digital signals from the microwave to the optical domain for a wide range of applications in both classical and quantum information processing. These span from developing transceivers for fiber-optic communication systems [1, 2] to radio-over-fiber applications in microwave photonics [3]. Recently, their performance (propagation loss and electro-optic coupling strength) has been improved to the point that they are leading candidates for building efficient microwave to optical photon transducers [4, 5], despite the $\approx 10^5\times$ difference between the wavelengths of the fields involved (cm for the microwave, µm for the optical) [6–8].

Both historically and recently [1, 2], state-of-the-art EOMs have been built around ferroelectric insulators [9] like lithium niobate [2], lithium tantalate [10] and barium titanate [11, 12] due to their high Pockels coefficient and low intrinsic optical absorption. On the other hand, ferroelectric insulators have certain intrinsic material limitations. These include long-term stability exemplified by the relaxation of the electro-optic response [6] and the resulting DC bias drift [1, 10], and inertness to reactive ion etching chemistries. The reliance on Ar-ion based physical etching techniques, with extensive sidewall redeposition and waveguide sidewall angles $\approx 60°$ [13] makes it difficult to leverage photonic bandgap structures [14] to shape and control waveguide dispersion [15, 16]. If we further desire that the material platform build on and leverage existing infrastructure investments in microelectronics [10] with a view towards scalability, integration with active electronics and long-term unit economic costs, then the choice can not be made based purely on de-

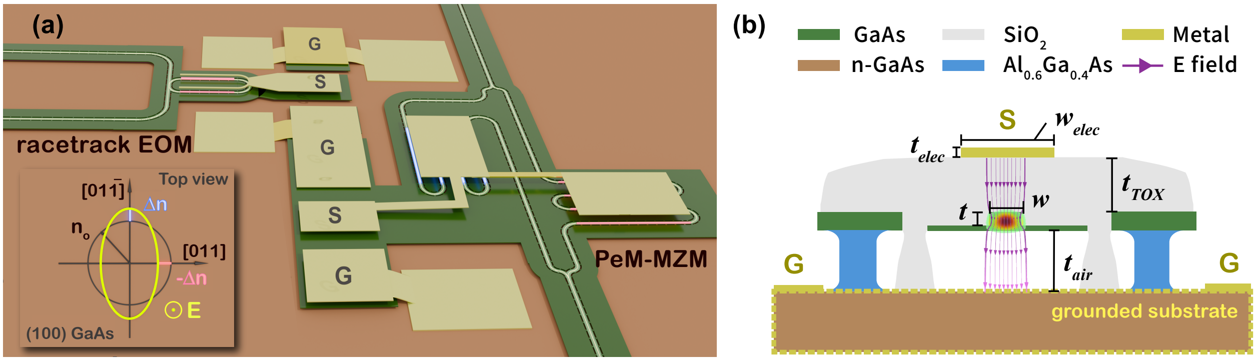

Fig. 1: (a) Schematic view of the suspended GaAs PIC platform showing the perpendicularly meandering Mach-Zehnder modulator (PeM-MZM, bottom) and racetrack resonator based EOM (top) on a (100) oriented GaAs wafer, showing the relative position between the electrodes and the underlying waveguides. The inset shows the planar projection of the GaAs index ellipsoid. Without an applied electric field along the [100] axis, GaAs is optically isotropic in-plane (black circle). When an external electric field is applied along the [100] axis, the ellipsoid deforms (yellow ellipse) with major and minor axes along the [011] or $[01\bar{1}]$ directions. Key for the PeM-MZM push-pull operation is that the refractive index change is equal and opposite in the two directions. (b) 2D cross section of suspended GaAs rib waveguide showing the interaction between the propagating optical field (transverse electric mode field calculated using FEM is overlaid to scale) and the out of plane DC / RF field (purple streamlines). Device parameters used in the simulations: waveguide width $w = 540\,\mathrm{nm}$, rib etch depth $t = 240\,\mathrm{nm}$, top oxide thickness $t_{\mathrm{TOX}} = 2.2\,\mu\mathrm{m}$, $Al_{0.6}Ga_{0.4}As$/air gap thickness $t_{\mathrm{air}} = 2\,\mu\mathrm{m}$, electrode thickness $t_{\mathrm{elec}} = 460\,\mathrm{nm}$, top electrode width $w_{\mathrm{elec}} = 5\,\mu\mathrm{m}$. The different components in the device are shown in the legend. The linear EO effect induces a refractive index change of $\Delta n_{\mathrm{eff}} = 1.279 \times 10^{-6}\,\mathrm{V}^{-1}$ in the GaAs waveguide due to the applied electric field.

vice metrics. This is best illustrated by the fact that modern data centres rely heavily on silicon photonics based transceivers [17], even though their individual device performance lags far behind state-of-the-art lithium niobate (LN) devices.

These factors make it interesting to continuously push the performance of EOMs fabricated in semiconductor platforms, in complement to efforts on ferroelectric insulators. Indium phosphide (InP) has been the traditional material of choice mainly due to the prospect of being able to monolithically integrate lasers on the same die [18] , and there have been some exciting recent developments on increasing component performance and integration density by moving towards InP-membrane on silicon technology [19–21]. ~~But, if we take the silicon photonics example above and consider the question of leveraging existing infrastructure investments, we argue that gallium arsenide (GaAs) presents a more logical choice to make the silicon-like electronics to photonics manufacturing leap by building on existing GaAs foundry investments~~ [22]. We focus instead on GaAs with a view towards leveraging extensive existing GaAs microelectronics foundry investments [22] in a silicon-like electronics to photonics transition, but note that the ideas developed here are equally applicable to InP. GaAs EOMs have a long and distinguished history [23, 24] and have found a niche in space-based (satellite) applications [25] where GaAs' radiation hardness and space qualification (from the electronics side) give it a significant advantage. ~~over other material platforms.~~

In addition to potential (electronic) foundry compatibility, another major driver for the pursuit of efficient GaAs EOMs is that the Ga(Al,In)As material system is the most extensively studied and well-developed for hosting quantum confined structures, in particular quantum dots and wells. InAs based quantum dots [26] hosted in a GaAs lattice currently provide the brightest solid-state single photon sources [27], and are currently the leading candidate for generating cluster states [12, 28] necessary for photonic implementations of measurement based quantum computing (MBQC). Implementing feedforward operations [12, 28] in MBQC architectures places a premium on integrated high-performance EOMs.

Despite their long development history, GaAs based EOMs have shared some common themes. They have generally relied on vertical epitaxially grown p-i-n diodes [24] which are reverse biased for the EO effect. To reduce free carrier absorption and also to account for the weak index contrast between GaAs core and AlGaAs cladding layers ($\Delta n \approx 0.2$), the mode sizes are typically $\approx 3\,\mu\mathrm{m}$ and the bend radii $> 100\,\mu\mathrm{m}$ which limits the component density. Given that the refractive index of GaAs is comparable to Si at telecommunication wavelengths [29], one should ideally be able to get silicon-like component density with the added benefit of high-performance EOMs by increasing the index

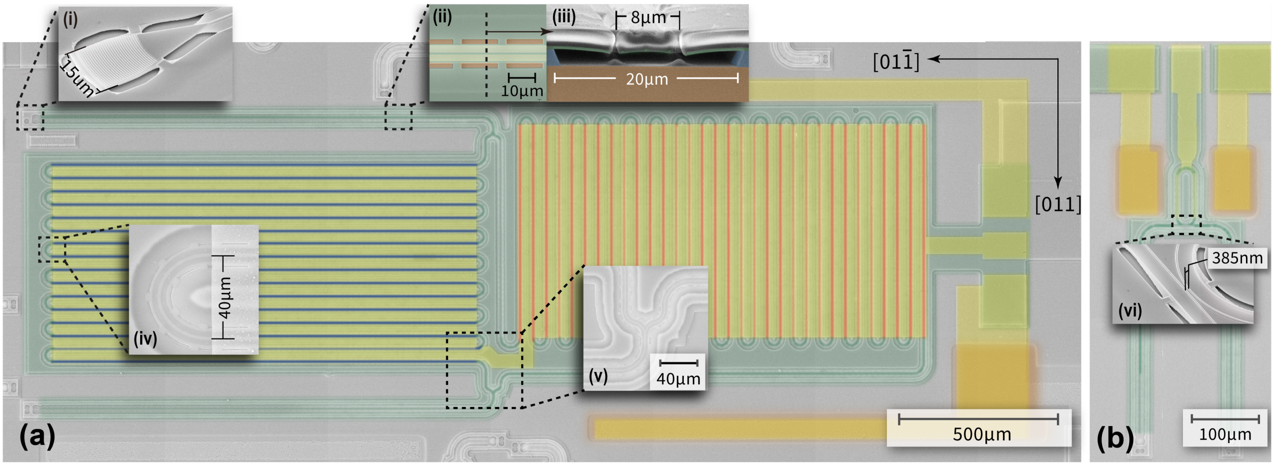

Fig. 2: False-colored SEM view of suspended GaAs (a) PeM-MZM and (b) racetrack EOM devices. The electrodes (yellow) covers the GaAs waveguide (green) with MZ arms meandering along [011] (red highlight) and [01$\bar{1}$] (blue highlight) directions. The uncolored regions represents GaAs substrate covered by deposited SiO_2 layer. Etch windows on SiO_2 layer are opened adjacent to the devices exposing bottom doped substrate (orange), allowing ground electrodes to form ohmic contact with substrate. Insets (i-vi) show zoomed views of key individual device components making up the EOM: (i) $15\,\mu m$-wide surface-normal grating coupler, (ii) top view of the rib waveguide suspended by $19\,\mu m$ spaced tethers, (iii) rib waveguide cross section showing a $20\,\mu m$-wide air gap opened beneath the waveguide, (iv) Euler U-shape bend with bend width of $40\,\mu m$ to mitigate bending loss, (v) 1-to-2 Y-splitter, (vi) bus waveguide-resonator coupler for the racetrack EOM with a coupling gap of $385\,nm$. SEM insets (i, ii, vi) are taken before capping the oxide, to give a clearer view of optical components and their suspension.

contrast, either via suspension [30] or by working with a gallium arsenide on an insulator platform using either wafer bonding [31] or membrane transfer [32]. The question of whether to use suspensions or wafer bonding to build high-performance GaAs devices is an open one and in many ways mirrors the debate in the LN EOM community [33]. We take the view that if bonding (and substrate removal) can be avoided without compromising device performance [34] and reliability, then one should do so. Moreover, suspended platforms (and incorporating MEMS-based approaches) have natural advantages whenever opto-mechanical interactions [35] are involved, such as in building microwave to optical quantum transducers [5] using acoustics [30] as an intermediary.

We illustrate the benefits of strong (sub-µm) confinement and the resultant reduction in device footprint by demonstrating true cm-scale push-pull modulators in GaAs. To clarify, by *true*, here we are referring to modulators analogous to X-cut LN [2], wherein the same voltage is applied to the two arms of the phase modulator, configured as a Mach-Zehnder interferometer, but one gets equal and opposite phase shifts. Unlike the X-cut LN case, which relies on lateral (in-plane) fields by exploiting the Pockels r_{33} coefficient and allows the signal electrode to be located at the centre of two outer ground planes, in GaAs, the Pockels r_{41} coefficient requires a vertically oriented field (as illustrated in Fig.1(a,b)) which results in equal phase shifts in the two parallel MZI arms. To build an EOM, therefore, one needs to apply RF signals anti-phase to the two MZI arms in a centre-tapped configuration (series push-pull) which requires additional bias and DC-decoupling circuitry [36]. To work around the issue in GaAs [37, 38], we use the fact that the application of a vertical electric field (along the [100], z-axis) breaks the in-plane refractive index symmetry. Light that is propagating along the [011] crystal axis picks up an equal and opposite phase shift to that propagating along the [01$\bar{1}$] axis (assuming transverse electric polarization, TE mode).

This is illustrated by the (in-plane) index ellipsoid shown in the inset of Fig.1(a) for one polarity of the vertical electric field. The ellipsoid will flip from being oblate to prolate as the field switches polarity. By folding the waveguide in the two arms of the MZI to lie (predominantly, ignoring the bends) along the [011] and [01$\bar{1}$] axes respectively, one achieves equal and opposite phase shifts in the two arms. This design is enabled primarily by the strong index contrast ($\Delta n \approx 2$) enabled by waveguide suspension, which allows tight folding, while maintaining a compact on-chip footprint. Building high-performance EOMs while working with the low r_{41} coefficient of GaAs requires cm-scale arm

lengths, which we demonstrate below, showing how far MEMS based ideas can be used to push integrated photonics platforms.

2 Device design and fabrication

Fig.1(a) shows a schematic of our proposed devices. The perpendicularly meandering Mach-Zehnder modulator (PeM-MZM) with the two waveguide arms oriented along the [011] and [01$\bar{1}$] respectively is indicated. Application of a vertical electric field (an FEM simulation of the electric field lines are shown in Fig.1(b)) breaks the in-plane refractive index symmetry and the (in-plane) index ellipsoid is oriented as shown in the figure inset. Given that GaAs is a zincblende crystal with symmetry group ($\bar{4}3m$), the change in refractive index (Δn) due to the linear electro-optic effect using the Pockels r_{41} coefficient, under the action of a vertically applied electric field can be written as:

$$\Delta n_{[011]} = +\frac{1}{2} n_o^3 r_{41} E_{\perp,[100]} \tag{1}$$

$$\Delta n_{[01\bar{1}]} = -\frac{1}{2} n_o^3 r_{41} E_{\perp,[100]} \tag{2}$$

where n_o is the GaAs refractive index (3.37 at 1550 nm), r_{41} = -1.5 pm V^{-1} is the relevant Pockels coefficient for the electro-optic interaction with a transverse electric (TE) polarized optical mode in the waveguide and a vertically oriented ($E_{\perp,[100]}$) electric field (either DC or RF). The equal and opposite signs of the refractive index change along the two crystal axes lies at the heart of the push-pull effect exploited in the PeM-MZM device. There is an additional quadratic EO effect, which is both significantly smaller, but more importantly gives equal phase shifts in the two arms, hence cancels out in this differential scheme. In theory, for the same applied electric field strength at the waveguide location, the refractive index change for GaAs based devices is ≈ 5× smaller than equivalent LN devices. To calibrate the push-pull effect and quantify the field strengths in the suspended waveguide platform, we also fabricate racetrack microring resonator based EOMs in the same platform where the sides of the racetrack are oriented along the crystal axes as shown in Fig.1(a), although here the quadratic EO contribution doesn't cancel out.

The devices are fabricated on an undoped 340 nm GaAs membrane which is released by undercutting an underlying $Al_{0.6}Ga_{0.4}As$ buffer layer using hydrofluoric acid (HF). While we chose to demonstrate the orientation dependent push-pull effect with bare GaAs in this work, these ideas can be extended to optimally doped p-i-n structures [37] with quantum wells, which would significantly enhance the modulation efficiency. The fabrication of the GaAs PIC follows a process similar to our previous work [29, 30, 34]. The suspended waveguide platform is encapsulated in silicon oxide deposited by plasma enhanced chemical vapor deposition. The oxide locks the structure mechanically providing rigidity [29], and also serves to offset the signal electrode from the waveguide layer (cf. Fig.1(b)). To build EOMs, we open up windows in the oxide layer to define the signal and ground electrodes and define the contacts using lift-off with an additional aligned lithography step. The r_{41} coefficient requires a vertically oriented electric field for operation. Therefore, the signal contact is deposited on top of the waveguide (offset by the oxide thickness ≈ 2 µm). To get the bottom contact underneath the waveguide to maximize the verticality of the dropped RF field (see Fig.1(b) for an FEM simulation showing the electric field lines around the waveguide), we use an n-doped GaAs substrate (1×10^{18} cm^{-3}) and use an annealed AuGe/Ni/Au metal stack to get an ohmic contact, see supplementary information (SI) section 1 for further details.

Fig.2(a,b) show false-colored SEM images of the PeM-MZM and racetrack EOM devices respectively. The different components of the device are shown by zoomed-in images added to the figure inset. Light is coupled onto and off the chip using focusing grating couplers (Fig.2(i)) and routed using suspended rib waveguides (Fig.2(ii, iii)). For the PeM-MZM designs, we split the light into the two MZ arms at the input using a Y-coupler (Fig.2(v)) and we use an identical Y-coupler at the output to recombine the light from the two arms. The push-pull effect originates from the orientation of the waveguide arms along two orthogonal axes as shown in the figure. The high refractive index contrast and strong mode confinement allows us to tightly fold the MZM. We use Euler bends [39] with effective bend radii of 20 µm (Fig.2(iv)) to ensure minimal mode mismatch between the straight and bent waveguide regions.

The PeM-MZM shown in Fig.2(a) are designed with arm lengths of 2.5 cm and 2.36 cm for the beam paths oriented along the [011] and the [01$\bar{1}$] axes respectively. We work with an asymmetric MZI design in these first-generation devices as it helps ease constraints on the layout and the spectral dependence on

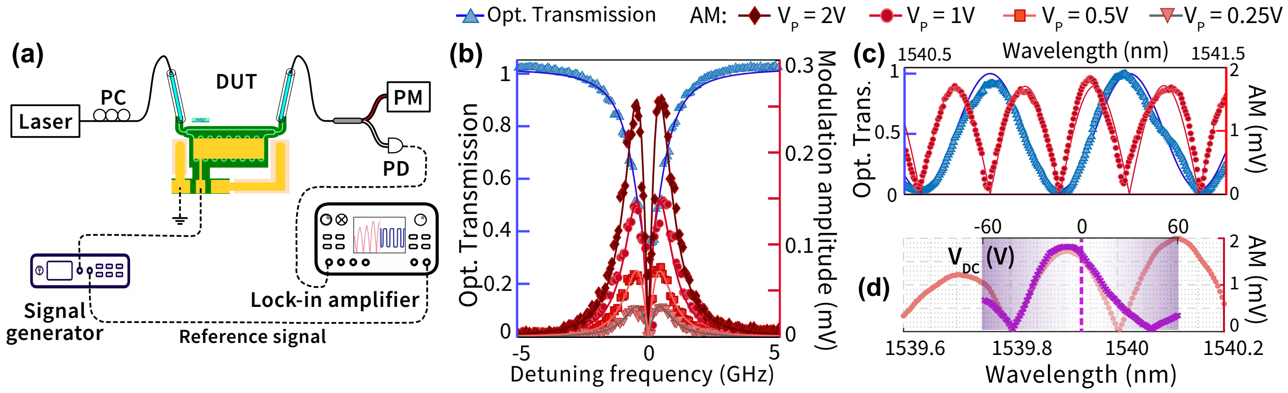

Fig. 3: (a) Experimental setup used for electro-optic modulation characterization. (b) A representative mode from the normalized optical transmission spectrum (blue) of the racetrack EOM, showing a loaded quality factor $Q \sim 1.47 \times 10^5$ and extinction depth $ER = 4.83\,\mathrm{dB}$ (SI, section 3). The measured spectrum is fit using a Lorentzian lineshape (blue, solid). The measured modulation amplitude (lock-in signal) is shown (red, scatter) and the predicted fit is shown in shades of red for different applied modulation voltages ranging from $0.25\,\mathrm{V}$ to $2\,\mathrm{V}$. We can see that the measured AM signal is clearly linear within this range. (c) A representative section of the optical transmission spectrum and modulation amplitude spectrum of the PeM-MZM device from Fig.2(a). The optical spectrum (blue scatter, normalized) is fitted with a sinusoidal curve (blue line), while the AM spectrum (red scatter) is fitted with a half-wave rectified sinusoidal model (red line). See SI section 4 for details on the fitting procedure. (d) AM spectrum of the PeM-MZM device measured with $0\,\mathrm{V}$ DC bias and $1\,\mathrm{V}$ RF voltage amplitude (pink scatter). Overlaid purple crosses show the AM response as the DC bias is swept from $-60\,\mathrm{V}$ to $60\,\mathrm{V}$ (top x-axis) with the laser wavelength parked at the dashed line, and the RF signal amplitude fixed at $1\,\mathrm{V}$. We believe the non-alignment of the data near λ=$1540\,\mathrm{nm}$ is due to temperature induced spectral shifts during data acquisition.

transmission helps us bound the losses of internal components like grating couplers, bends and Y-splitters. By optimizing the layout, the meandering arm lengths can in principle be made symmetric. The overall design takes up an on-chip footprint of $1\,\mathrm{mm} \times 3.1\,\mathrm{mm}$. We were conservative in our designs with respect to lateral undercut provision and the radii of the Euler bends to ensure working devices in these first generation experiments. ~~By optimizing both parameters, we expect to see a further $2-5\times$ reduction in device footprint.~~

The scale of the device in Fig.2(a) clearly shows the potential of incorporating MEMS based techniques into integrated photonics platforms [40], beyond silicon wherein thin film on-insulator substrates are not readily available or are limited in substrate size. We maintain sub-µm mode confinement over 2.5 cm scale on-chip path lengths, and the platform is stable to enable sensitive on-chip interferometry. To ease the fabrication constraints in these proof-of-principle devices, we chose to work with lumped electrodes for the EOMs, shown schematically in Fig.1(a), and indicated by the gold pads in Fig.2(a,b) for the PeM-MZM and the racetrack EOM respectively. For the PeM-MZM device in Fig.2(a), the electrode overlaps 2.08 cm of the folded waveguide in both arms to maintain the symmetry of the push-pull operation.

3 Device characterization

We characterize linear electro-optic modulation in our devices using the setup shown in Fig.3(a). Light from a tunable laser (Santec, TSL-550) is coupled into and out of the device under test (DUT) from a fiber array using grating couplers. As the laser wavelength is scanned, a modulation (AC) signal of frequency 1 MHz, and peak amplitude 1 V for PeM-MZM (0.25 V-2 V for the racetrack EOM) is applied to the ground-signal-ground electrode configuration using a microwave probe. The transmitted optical signal is measured using both an optical power meter (Thorlabs, PM100USB) to record the transmission spectrum, and with a high-speed photodiode (Optilab, APR-10-MC), whose output is fed into a lock-in amplifier (Stanford Research Systems, SR865A) for modulation amplitude measurement. The signal generator (Tektronix, AFG2021) provides the reference signal for the lock-in, as indicated in Fig.3(a). The phase modulation induced by the EO effect is translated to amplitude modulation (AM) by the spectral dependence of the DUT transmission, and this translated AM is recorded as the modulation amplitude by the lock-in amplifier from the photodiode output.

Fig.3(b,c) shows the measured modulation amplitude spectra overlaid on the optical transmission spec-

tra for the racetrack EOM and the PeM-MZM devices respectively. The measured modulation amplitude as a function of laser wavelength agrees well with the gradient of the optical transmission spectra, in line with the PM to AM translation argument discussed above. Fitting the modulation amplitude (see SI section 4 for details) allow us to extract the modulation efficiency, expressed as a spectral tunability (η, [pm V^{-1}]) or an equivalent half-wave voltage (V_π) need to shift the transmission from a maxima to a minima (or vice-versa). For racetrack EOMs with a loaded quality factor $Q \approx 1.47{\times}10^5$ and extinction ratio $ER =$ 4.83 dB, we extract an η =(0.351 $\pm$ 0.008) pm V^{-1} and a V_π =(31.9 $\pm$ 0.8) V. For the PeM-MZM devices, the values are η =(0.139 $\pm$ 0.003) pm V^{-1} and a V_π =(54.3 $\pm$ 1.3) V. The V_π for PeM-MZM can also be directly quantified through a DC sweep measurement, as shown in Fig.3(d). Here, we repeat the modulation experiment as in Fig.3(a), but add a DC bias voltage on top of the AC voltage (amplitude = 1 V). By sweeping the DC bias voltage, one can in principle traverse the optical transmission spectrum, as shown in Fig.3(d), and read out the V_π directly. The racetrack EOM measurement serves as a reference for the more complex PeM-MZM devices. From the modulation measurements, we can extract an equivalent refractive index change per unit applied voltage for both devices. This gives us Δn_{eff} = 1.084 $\times$ $10^{-6}\mathrm{V}^{-1}$ for racetrack EOM and $\Delta n_{\mathrm{eff}} = 6.97 \times 10^{-7}\mathrm{V}^{-1}$ for PeM-MZM. The extracted Δn_{eff} for the racetrack EOM agrees well with the predicted $\Delta n_{\mathrm{eff}} = 1.279{\times}10^{-6}\mathrm{V}^{-1}$ using FEM simulation (cf. SI section 2).

We can also demonstrate the opposite phase shifts along the [011] and [01$\bar{1}$] axes by designing unbalanced MZMs with only a single arm (SeM-MZM) meandering along the respective crystal axes, as shown in Fig.4(i, ii). The meandering arm lengths are kept identical in both devices and their nominal optical transmission spectra are similar (as shown Fig.4(a)). By parking the laser at the mid-point of the amplitude modulation spectrum (shown by the dashed lines in Fig.4(a)) and applying a DC voltage sweep of fixed polarity (0 V-32 V), we see that the differential change in modulation amplitude is opposite with DC bias. This is because the underlying MZI transmission spectrum is either red or blue detuned in the two cases, depending on waveguide orientation. Fig.4(b) plots the measured (differential) modulation amplitude, from the mid-point, as the applied DC bias is increased from 0 to 32 V. The push-pull effect can clearly be seen. While the opposite nature of the effect in the two arms is easy to verify using Fig.4, the effect being exactly equal in magnitude is more challenging to quantify, given the variability between devices. We can in turn bound the difference between the two arms by quantifying the V_π of the two SeM-MZM devices, which were designed to have the same meandering arm path lengths. We extract the two V_π to be, respectively 86 V for device (i) and 93 V for device (ii).

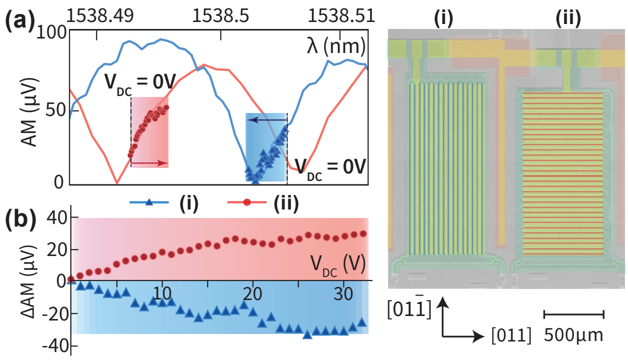

Fig. 4: Control experiments to demonstrate the push-pull nature of the effect: DC bias induced phase shift on two SeM-MZM devices, with a single arm meandering along [011](i) or [01$\bar{1}$](ii) direction. The meandering arm lengths are designed to be nominally equal in the two cases. (a) AM spectra for SeM-MZMs driven by a 1 MHz modulation signal of amplitude 1 V, (blue for (i), red solid for (ii)). Overlaid scattered plot (red circles and blue triangles) shows the shift in the AM spectrum when the DC bias voltage is swept from 0 V (black dashed line) to 32 V. The laser wavelength is indicated by the dashed line (b) Replotting the data from (a) to show the differential AM change as a function of applied DC bias voltage. The differential shift (ΔAM = AM(V_{DC})-AM(0)) is plotted with reference to the zero DC bias point. The opposite slopes of the differential AM voltage with respect to the bias voltage V_{DC} clearly shows the push-pull effect in action.

We measure the modulation bandwidth (BW) of the racetrack EOM and the PeM-MZM devices using a modified version of the setup shown in Fig.3(a). Here, we use a vector network analyzer (VNA, R&S ZVL) to drive (via Port 1) the device under test with a microwave signal (0 dBm, 225 mV RMS) and sweep the modulation frequency from 100 MHz to 9 GHz. The modulated signal is measured using a high-speed amplified photodiode (Optilab, APR-10-MC) whose output is fed back into the VNA (port 2) to perform a standard EO S_{21} measurement. Fig.5(a) plots the normalized electro-optic frequency response of the racetrack (brown) and PeM-MZM (green) devices. The device response is normalized to 100 MHz, cf. SI section 5 for details on the normalization procedure. The extracted 3 dB modulation bandwidths of the racetrack EOM and PeM-MZM devices are $\approx$ 2 GHz and

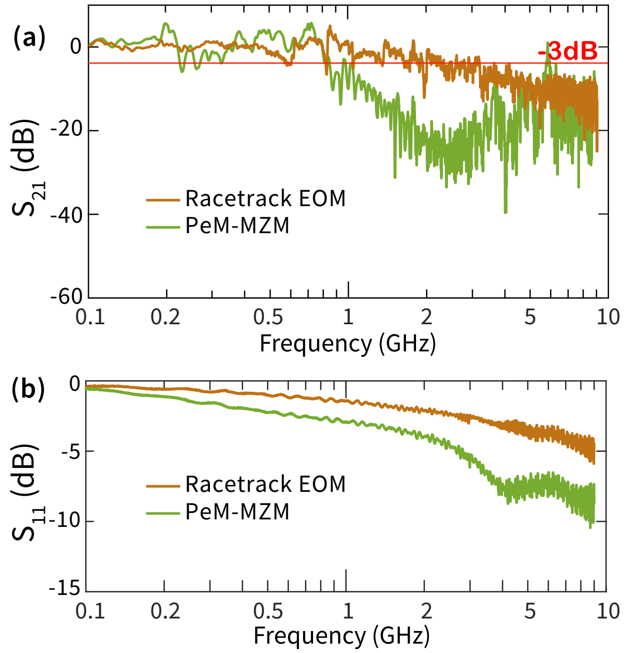

Fig. 5: (a) Measured (normalized) EO frequency response (S_{21}) for 2 cm long PeM-MZM (green) and racetrack EOM ring modulator (brown). The frequency response is normalized to 100 MHz and the normalization procedure is outlined in SI section 5. (b) Measured electrode reflection spectra (S_{11}) for the devices. The wiggles apparent in the bandwidth spectra (S_{21}) occur due to a combination of electrode reflection and photodiode gain normalization.

≈ 0.8 GHz respectively. In these proof-of-principle devices, the electrodes (see Fig.1(a)) were not optimized for high-speed operation, but more to ease fabrication constraints in order to demonstrate the push-pull effect in cm-scale devices. Therefore, our BW is primarily limited by the RC time constant of these lumped element electrodes. Fig.5(b) plots the measured electrode reflection S_{11} spectra for the racetrack resonator and the PeM-MZM device. The wiggles apparent in the measured bandwidth (S_{21}) spectrum originate from a combination of the bare electrode response and the normalization procedure detailed in SI section 5. SI section 5 shows the extracted amplified photodiode gain spectrum which is non-monotonic and has an impact on the measured bandwidth spectrum.

4 Discussion

While the results outlined in this paper clearly demonstrate the orientation dependent push-pull effect in the PeM-MZM devices, and the scale (2 cm suspended arm lengths in the MZI) shows the promise of bringing MEMS based nanofabrication approaches to integrated photonics platforms, the actual device performance leaves some scope for improvement. Many of the limitations in the EOM performance metrics outlined above can be traced to conservative design choices made on the nanofabrication side to get working devices. As noted above, the scale of these devices far exceeds what has been previously demonstrated in a suspended GaAs PIC platform [29, 30], coupled with the additional metallization constraints to generate the vertical field required at the waveguides.

Below, we outline how the various components of the PeM-MZM can be improved to achieve state-of-the-art modulator performance [23, 24], keeping in mind the trade-offs between increased device complexity and reduced fabrication yield. The three main components to improve are the underlying passive optical performance (insertion and propagation loss), improving the modulation efficiency and increasing the modulation bandwidth. We consider each in turn.

While we are clearly able to demonstrate the orientation-dependent push-pull effect using the PeM-MZM devices and achieve working EOMs, the underlying passive device optical performance needs improvement. In the device shown in Fig.2(a), we measure an end-to-end insertion loss of 29.8 dB, which we can subdivide into 7.0 dB per grating coupler (2×), 1.0 dB per

Y-splitter (2×) and 13.8 dB of propagation loss. SI section 3 provides further details on the loss extraction of the individual components. The optical propagation loss of 5.5 dB cm^{-1}, extracted from the loaded quality factor of the racetrack resonators fabricated on the same chip, is 2.3× greater than the 2.4 dB cm^{-1} [34] that we have demonstrated in purely passive devices before.

The excess loss in the grating coupler is mainly due to an incomplete undercut of the underlying AlGaAs buffer layer. As noted in the fabrication procedure (SI section 1), we rely on a timed HF acid etch to remove the AlGaAs layer and suspend the waveguides. Given the lack of tensile stress in the GaAs device layer, overetching the buffer layer causes the membranes to sag [29] and given the scale of the devices (2.5 cm in each arm and 2 cm suspended sections), we were keen to prevent waveguide collapse with a view towards getting functional devices. Therefore, we restricted the (over)-etch time, and that resulted in an incomplete undercut of the AlGaAs sacrificial layer with the worst affected location being the grating coupler on account of its size, more specifically, the distance from the centre of the component to the nearest etch window. With process optimization, we should be able to achieve the loss metrics we have previously demonstrated [34] on these cm-scale devices. Moving to wider waveguide widths (≈750 nm) is a potential solution as it reduces surface loss while maintaining single-mode operation, although it comes at the cost of device footprint as the minimum bend radius increases from ≈ 10 µm to ≈ 20 µm as the waveguide width is increased from 550 nm to 750 nm.

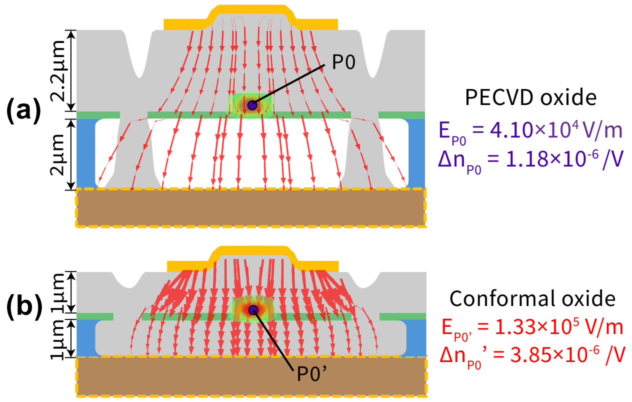

Fig. 6: Electric field distribution comparison of the suspended GaAs waveguide devices shown in this work (a) with the proposed optimized geometry (b). Both the top and bottom cladding spacing to the electrodes can be reduced from ≈ 2 µm in the current devices to 1 µm without affecting optical performance. More importantly, by using conformal PECVD oxide deposition, the field strength at the waveguide (and the associated index change) can be significantly improved, as discussed in the main text. The FEM simulation of the local electric field strength is overlaid with optical mode and depicted using arrowheads that are scaled proportionally. Point P_0 locates the center of waveguide.

The second area of improvement, is the optimization of top and bottom cladding thickness, and electrode design to maximize the refractive index change (Δn) per unit applied voltage and therefore maximize the modulation efficiency. In a vertical geometry like the GaAs EOM, the device can be approximated, to first order, as a series of three capacitors with dielectric constants roughly corresponding to the top cladding, waveguide and bottom cladding respectively. The voltage drop for such a series capacitor configuration scales inversely with the dielectric constant, which means a significant fraction of the field drops across the bottom air cladding. Both the top and bottom cladding thickness can be reduced by half to 1 µm from the current devices without affecting optical performance significantly, and ensuring higher electric field strengths for a given applied voltage. By moving to a top and bottom oxide cladding using conformal PECVD [41], we can improve the electric field strength by ≈ 3.3× and the overall Δn by ≈ 3.3×, cf. Fig.6. By building the same 2 cm PeM-MZM devices, we expect a $V_\pi \approx 9.0$ V. We would like to emphasize here that this optimization is performed keeping the GaAs device layer thickness fixed at 340 nm in keeping with standard silicon photonics foundry offerings. Increasing the thickness to 500 nm brings the V_π down to ≈ 5.5 V for similar length devices. We would like to note that moving to

a conformal PECVD reduces the index contrast and therefore mode confinement slightly, but this effect is very small in comparison to the increased field strength and associated increased modulation efficiency.

The final area of improvement to the devices reported in this work is incorporating travelling wave electrodes around the waveguides and velocity matching the microwave and optical fields with a view towards increasing the operational bandwidth. While the design of travelling wave electrodes is well-understood for GaAs [24, 42], adapting these designs to our tightly folded geometries while maintaining a low microwave insertion loss will require a re-optimization of the optical and microwave performance to maximize the device figure of merit. A second fabrication challenge that needs to be addressed is the thickness of the metal electrodes. To reduce the resistive loss at high frequencies, the metal thickness needs to be $> 500\,\mathrm{nm}$, and the compatibility of such a dense metal stack with a suspended waveguide platform needs to be demonstrated in practice.

5 Conclusions

We have demonstrated *true* push-pull electro-optic modulators in a suspended GaAs PIC platform by exploiting the orientation induced asymmetry of the Pockels r_{41} coefficient and folding the two arms of an MZI along orthogonal crystal axes ([011] and $[01\bar{1}]$, respectively). We also show that sub-μm mode confinement can be maintained across cm-scale devices in a suspended platform with relatively high-performance. This work provides a proof-of-principle demonstration of the idea of using geometry to exploit tensorial coefficients in crystalline media, mainly compound semiconductors, and serves as a building block for engineering quasi-phase matched interactions in curvilinear geometries in materials with $\bar{4}$ crystal symmetry [43]. By pushing on the surface loss frontier through improved surface passivation [34], these devices can potentially approach the regime of *mesoscopic* nonlinear optics [44]. As outlined in the introduction, semiconductor based EOMs have certain unique advantages over traditional ferroelectric insulators, but realizing these benefits, especially from a systems perspective, requires a coordinated effort on the photonics, microwave, materials and manufacturing fronts.

Acknowledgment: We would like to thank Laurent Kling, Stephen Clements, Robert Walker, Ian Farrer, Edmund Clarke and Andrew Murray for helpful suggestions and feedback.

Research funding: We gratefully acknowledge funding support from the UK's Engineering and Physical Sciences Research Council (GASP, EP/V052179/1) and the European Research Council (ERC-StG, SBS 3-5, 758843). Nanofabrication was carried out using equipment funded by an EPSRC capital equipment grant (QuPIC, EP/N015126/1). The GaAs wafers were sourced from the UK's national epitaxy facility in Sheffield (EP/X015300/1).

Author contributions: All authors have accepted responsibility for the entire content of this manuscript and approved its submission.

Conflict of interest: Authors state no conflict of interest.

Data availability statement: Data underlying the results presented in this paper are not publicly available at this time but may be obtained from the authors upon reasonable request.

References

[1] E. L. Wooten, K. M. Kissa, A. Yi-Yan, E. J. Murphy, D. A. Lafaw, P. F. Hallemeier, D. Maack, D. V. Attanasio, D. J. Fritz, G. J. McBrien, *et al.*, "A review of lithium niobate modulators for fiber-optic communications systems," *IEEE Journal of selected topics in Quantum Electronics*, vol. 6, no. 1, pp. 69–82, 2000.

[2] M. Zhang, C. Wang, P. Kharel, D. Zhu, and M. Lončar, "Integrated lithium niobate electro-optic modulators: when performance meets scalability," *Optica*, vol. 8, no. 5, pp. 652–667, 2021.

[3] J. Capmany and D. Novak, "Microwave photonics combines two worlds," *Nature photonics*, vol. 1, no. 6, p. 319, 2007.

[4] X. Han, W. Fu, C.-L. Zou, L. Jiang, and H. X. Tang, "Microwave-optical quantum frequency conversion," *Optica*, vol. 8, no. 8, pp. 1050–1064, 2021.

[5] K. C. Balram and K. Srinivasan, "Piezoelectric optomechanical approaches for efficient quantum microwave-to-optical signal transduction: the need for co-design," *Advanced Quantum Technologies*, vol. 5, no. 3, p. 2100095, 2022.

[6] J. Holzgrafe, N. Sinclair, D. Zhu, A. Shams-Ansari, M. Colangelo, Y. Hu, M. Zhang, K. K. Berggren, and M. Lončar, "Cavity electro-optics in thin-film lithium niobate for efficient microwave-to-optical transduction," *Optica*, vol. 7, no. 12, pp. 1714–1720, 2020.

[7] T. P. McKenna, J. D. Witmer, R. N. Patel, W. Jiang, R. Van Laer, P. Arrangoiz-Arriola, E. A. Wollack, J. F. Herrmann, and A. H. Safavi-Naeini, "Cryogenic microwave-to-optical conversion using a triply resonant lithium-niobate-on-sapphire transducer," *Optica*, vol. 7, no. 12, pp. 1737–1745, 2020.

[8] H. K. Warner, J. Holzgrafe, B. Yankelevich, D. Barton, S. Poletto, C. Xin, N. Sinclair, D. Zhu, E. Sete, B. Langley, *et al.*, "Coherent control of a superconducting qubit using light," *arXiv preprint arXiv:2310.16155*, 2023.

[9] T. A. Maldonado, "Electro-optic modulators," *Handbook of optics*, vol. 2, pp. 13–11, 1995.

[10] C. Wang, D. Fang, J. Zhang, A. Kotz, G. Lihachev, M. Churaev, Z. Li, A. Schwarzenberger, X. Ou, C. Koos, *et al.*, "Ultrabroadband thin-film lithium tantalate modulator for high-speed communications," *Optica*, vol. 11, no. 12, pp. 1614–1620, 2024.

[11] S. Abel, F. Eltes, J. E. Ortmann, A. Messner, P. Castera, T. Wagner, D. Urbonas, A. Rosa, A. M. Gutierrez, D. Tulli, *et al.*, "Large Pockels effect in micro-and nanostructured barium titanate integrated on silicon," *Nature materials*, vol. 18, no. 1, pp. 42–47, 2019.

[12] K. Alexander, A. Bahgat, A. Benyamini, D. Black, D. Bonneau, S. Burgos, B. Burridge, G. Campbell, G. Catalano, A. Ceballos, *et al.*, "A manufacturable platform for photonic quantum computing," *arXiv preprint arXiv:2404.17570*, 2024.

[13] F. Kaufmann, G. Finco, A. Maeder, and R. Grange, "Redeposition-free inductively-coupled plasma etching of lithium niobate for integrated photonics," *Nanophotonics*, vol. 12, no. 8, pp. 1601–1611, 2023.

[14] R. D. V. Meade, S. G. Johnson, and J. N. Winn, *Photonic crystals: Molding the flow of light*. Princeton University Press, 2008.

[15] K. Kawahara, T. Tsuchizawa, N. Yamamoto, Y. Maegami, K. Yamada, S. Hara, and T. Baba, "High-speed, low-voltage, low-bit-energy silicon photonic crystal slow-light modulator with impedance-engineered distributed electrodes," *Optica*, vol. 11, no. 9, pp. 1212–1219, 2024.

[16] A. Nardi, A. Davydova, N. Kuznetsov, M. H. Anderson, C. Möhl, J. Riemensberger, T. J. Kippenberg, and P. Seidler, "Integrated chirped photonic-crystal cavities in gallium phosphide for broadband soliton generation," *Optica*, vol. 11, no. 10, pp. 1454–1461, 2024.

[17] M. Glick, L. Liao, and K. Schmidtke, *Integrated Photonics for Data Communication Applications*. Elsevier, 2023.

[18] G. E. Hoefler, Y. Zhou, M. Anagnosti, A. Bhardwaj, P. Abolghasem, A. James, S. Luna, P. Debackere, A. Dentai, T. Vallaitis, *et al.*, "Foundry development of system-on-chip InP-based photonic integrated circuits," *IEEE Journal of Selected Topics in Quantum Electronics*, vol. 25, no. 5, pp. 1–17, 2019.

[19] T. Kakitsuka, K. Hasebe, T. Fujii, T. Sato, K. Takeda, and S. Matsuo, "Inp-based membrane optical devices for large-scale photonic integrated circuits," *NTT Technical Review*, vol. 13, no. 5, 2015.

[20] J. J. van der Tol, Y. Jiao, L. Shen, A. Millan-Mejia, V. Pogoretskii, J. P. van Engelen, and M. K. Smit, "Indium phosphide integrated photonics in membranes," *IEEE Journal of Selected Topics in Quantum Electronics*, vol. 24, no. 1, pp. 1–9, 2017.

[21] Y. Wang, Y. Jiao, and K. Williams, "Scaling photonic integrated circuits with inp technology: A perspective," *APL Photonics*, vol. 9, no. 5, 2024.

[22] Y.-C. Wang, "GaAs foundry technologies," in *Proceedings of Technical Program-2014 International Symposium on VLSI Technology, Systems and Application (VLSI-TSA)*, pp. 1–2, IEEE, 2014.

[23] N. Dagli, "Wide-bandwidth lasers and modulators for RF photonics," *IEEE Transactions on microwave theory and techniques*, vol. 47, no. 7, pp. 1151–1171, 1999.

[24] R. G. Walker, N. I. Cameron, Y. Zhou, and S. J. Clements, "Optimized gallium arsenide modulators for advanced modulation formats," *IEEE Journal of Selected Topics in Quantum Electronics*, vol. 19, no. 6, pp. 138–149, 2013.

[25] R. Walker, N. Cameron, Y. Zhou, C. Main, G. Hoy, and S. Clements, "50GHz gallium arsenide electro-optic modulators for spaceborne telecommunications," in *International Conference on Space Optics—ICSO 2018*, vol. 11180, pp. 2683–2691, SPIE, 2019.

[26] P. Lodahl, S. Mahmoodian, and S. Stobbe, "Interfacing single photons and single quantum dots with photonic nanostructures," *Reviews of Modern Physics*, vol. 87, no. 2, pp. 347–400, 2015.

[27] N. Tomm, A. Javadi, N. O. Antoniadis, D. Najer, M. C. Löbl, A. R. Korsch, R. Schott, S. R. Valentin, A. D. Wieck, A. Ludwig, *et al.*, "A bright and fast source of coherent single photons," *Nature Nanotechnology*, vol. 16, no. 4, pp. 399–403, 2021.

[28] J. R. Scott and K. C. Balram, "Timing constraints imposed by classical digital control systems on photonic implementations of measurement-based quantum computing," *IEEE Transactions on Quantum Engineering*, vol. 3, pp. 1–20, 2022.

[29] P. Jiang and K. C. Balram, "Suspended gallium arsenide platform for building large scale photonic integrated circuits: passive devices," *Optics express*, vol. 28, no. 8, pp. 12262–12271, 2020.

[30] A. Khurana, P. Jiang, and K. C. Balram, "Piezo-optomechanical signal transduction using lamb-wave supermodes in a suspended gallium arsenide photonic-integrated-circuit platform," *Physical Review Applied*, vol. 18, no. 5, p. 054030, 2022.

[31] E. J. Stanton, J. Chiles, N. Nader, G. Moody, N. Volet, L. Chang, J. E. Bowers, S. Woo Nam, and R. P. Mirin, "Efficient second harmonic generation in nanophotonic GaAs-on-insulator waveguides," *Optics express*, vol. 28, no. 7, pp. 9521–9532, 2020.

[32] G. Roelkens, J. Zhang, L. Bogaert, E. Soltanian, M. Billet, A. Uzun, B. Pan, Y. Liu, E. Delli, D. Wang, *et al.*, "Present and future of micro-transfer printing for heterogeneous photonic integrated circuits," *APL Photonics*, vol. 9, no. 1, 2024.

[33] S. Mookherjea, V. Mere, and F. Valdez, "Thin-film lithium niobate electro-optic modulators: to etch or not to etch," *Applied Physics Letters*, vol. 122, no. 12, 2023.

[34] R. Thomas, H. Li, J. Laverock, and K. C. Balram, "Quantifying and mitigating optical surface loss in suspended GaAs photonic integrated circuits," *Optics Letters*, vol. 48, no. 15, pp. 3861–3864, 2023.

[35] Ž. Zobenica, R. W. van der Heijden, M. Petruzzella, F. Pagliano, R. Leijssen, T. Xia, L. Midolo, M. Cotrufo, Y. Cho, F. W. Van Otten, *et al.*, "Integrated nano-opto-electro-mechanical sensor for spectrometry and nanometrology," *Nature communications*, vol. 8, no. 1, p. 2216, 2017.

[36] R. Walker, "High-speed electrooptic modulation in GaAs/GaAlAs waveguide devices," *Journal of lightwave technology*, vol. 5, no. 10, pp. 1444–1453, 1987.

[37] N. Dagli, "III-V compound semiconductor electro-optic modulators: Chapter 4," in *High-Speed Photonic Devices*, ch. 4, pp. 63–108, Taylor & Francis, 2007.

[38] L. Midolo, S. L. Hansen, W. Zhang, C. Papon, R. Schott, A. Ludwig, A. D. Wieck, P. Lodahl, and S. Stobbe, "Electro-optic routing of photons from a single quantum dot in photonic integrated circuits," *Optics Express*, vol. 25, no. 26, pp. 33514–33526, 2017.

[39] M. Cherchi, S. Ylinen, M. Harjanne, M. Kapulainen, and T. Aalto, "Dramatic size reduction of waveguide bends on a micron-scale silicon photonic platform," *Optics express*, vol. 21, no. 15, pp. 17814–17823, 2013.

[40] N. Quack, H. Sattari, A. Y. Takabayashi, Y. Zhang, P. Verheyen, W. Bogaerts, P. Edinger, C. Errando-Herranz, and K. B. Gylfason, "MEMS-enabled silicon photonic integrated devices and circuits," *IEEE Journal of Quantum Electronics*, vol. 56, no. 1, pp. 1–10, 2019.

[41] J. R. Abelson and G. S. Girolami, "New strategies for conformal, superconformal, and ultrasmooth films by low temperature chemical vapor deposition," *Journal of Vacuum Science & Technology A*, vol. 38, no. 3, 2020.

[42] J. Shin, C. Ozturk, S. Sakamoto, Y. Chiu, and N. Dagli, "Novel t-rail electrodes for substrate removed low-voltage high-speed GaAs/AlGaAs electrooptic modulators," *IEEE transactions on microwave theory and techniques*, vol. 53, no. 2, pp. 636–643, 2005.

[43] P. S. Kuo, J. Bravo-Abad, and G. S. Solomon, "Second-harmonic generation using-quasi-phasematching in a GaAs whispering-gallery-mode microcavity," *Nature communications*, vol. 5, no. 1, p. 3109, 2014.

[44] M. Jankowski, R. Yanagimoto, E. Ng, R. Hamerly, T. P. McKenna, H. Mabuchi, and M. Fejer, "Ultrafast second-order nonlinear photonics—from classical physics to non-gaussian quantum dynamics: a tutorial," *Advances in Optics and Photonics*, vol. 16, no. 2, pp. 347–538, 2024.
